# Cognitive dysfunction and dementia in movement disorders

**DOI:** 10.1590/s1980-5764-2016dn1004001

**Published:** 2016

**Authors:** Egberto Reis Barbosa, Hélio A. G. Teive, Vitor Tumas

**Affiliations:** Editors

Alzheimer's disease (AD) and Parkinson's disease (PD) represent the most common neurodegenerative disorders, and while AD is the most prevalent cause of dementia, PD, the commonest bradykinetic movement disorder, has a cumulative prevalence of dementia of 75-90% (PD dementia – PDD) particularly in patients with a disease duration of 10 years or longer. At the opposite end of the spectrum of movement disorders (MDs), namely the hyperkinetic MD syndromes, Huntington's disease (HD) is a progressive, fatal, neurodegenerative disorder, with autosomal dominant heritage, characterized by the presence of movement disorders (particularly chorea), behavior abnormalities and cognitive dysfunction, including dementia. Cognitive dysfunction or dementia is also common in several other MDs including atypical parkinsonism – such as progressive supranuclear palsy, dementia with Lewy bodies and corticobasal syndrome (CBS) – and hyperkinetic MDs, such as Wilson's disease (WD), hereditary spastic paraplegias (HSP) and spinocerebellar ataxias (SCA). More recently, autoimmune encephalitis, as well as paraneoplastic disorders, is known to present with a combination of MDs and neuropsychiatric syndromes with cognitive impairment.

In this issue of **Dementia & Neuropsychologia** we have published five reviews and 10 original papers about cognitive dysfunction and dementia in different MDs. **Teixeira et al.** present an updated review about cognitive and psychiatric features of HD in which the authors stated that the progression of cognitive impairment and neuropsychiatric symptoms occurs in parallel with neurodegeneration. Additionally, **Santoro et al.** wrote a fine review about cell-based technologies; including human fetal tissue, neural stem cells of human fetal tissue origin or embryonic stem cells, induced pluripotent stem cells, and mesenchymal stem cells; for the treatment of HD. **Parmera et al.** wrote an excellent paper on CBS, constituting a heterogeneous spectrum of different entities. **Faber et al.** evaluated cognitive dysfunction in HSP - which represents more than 70 different genetic subtypes - and other motor neuron disorders.

**Barbosa et al.** reviewed gait, posture and cognition in PD, emphasizing that cognition plays an important role for the loss of postural control, thus contributing to the increased risk of falls in these patients. **Machado et al.** evaluated the neuropsychological profile of PD patients selected for deep brain stimulation surgery, in which the authors studied 30 PD patients with surgical indication for DBS, using several instruments including the MMSE, FAB, MoCA, BDI, semantic verbal fluency, PDQ-39, PDSS, and the UPDRS and Hoehn-Yahr scales. They concluded that there was cognitive impairment in 56.7% of patients as measured by FAB and 76% by the MoCA, even in the early stages of the disease.

**Olchik et al.** evaluated the impact of cognitive performance on the quality of life of individuals with PD and observed a direct correlation between lower QOL scores and worse cognitive performance. **Souza et al.** conducted a cluster analysis of cognitive performance in a sample of 100 patients with PD and found 40 patients with PDD, 39 with PD MCI, and 21 with PD normal cognition. They concluded that cognitive impairment in PD occurs progressively and heterogeneously in most patients.

**Camargo et al.** investigated the perception of apathy by caregivers of patients with dementia in PD. They assessed 39 patients with PD, 97.4% of whom had results consistent with apathy, a disorder related to PDD. **Camargo et al.** also performed a comparison of the use of screening tools for evaluating cognitive impairment in 50 patients with PD, and found that 82% of the patients had dementia, and that the MoCA test had sensitivity of 87.80% and specificity of 88.89%. **Borges et al.** studied some aspects of the validity of the MoCA for evaluating cognitive impairment in 66 Brazilian patients with PD, using the MDS-UPDRS, Hoehn-Yahr stage and MoCA test: 19.7 % of patients were classified as cognitively normal, 36.3 % had MCI and 43.9 % dementia. The authors believe that some of the MoCA subtests may be difficult for PD patients with low educational level to complete, contributing to the test's poor diagnostic accuracy.

**Dias et al.** evaluated telerehabilitation in PD and its influence on cognitive status in a study of 50 patients with PD in stages 2-4 according to the Hoehn-Yahr scale, and found that 76% of patients would agree to participate in future telerehabilitation protocols, especially those with higher educational levels and better cognitive status.

**Moro & Teive** evaluated the neuropsychological findings in spinocerebellar ataxia type 10 and found that SCA10 patients had worse depression scores and cognitive performance when compared with healthy individuals, concluding that these patients have mild cognitive and mood dysfunctions. **Frota et al.** evaluated which factors are associated with global cognitive impairment in a group of 20 patients with WD using several cognitive assessment tests, and concluded that global cognitive impairment was common in this sample of patients and was associated with low educational level and brain MRI findings. **Simabukuro et al.** studied the importance of recognizing faciobrachial dystonic seizures in rapidly progressive dementias in a report of two Brazilian patients with faciobrachial dystonic seizures and rapid cognitive decline due to anti-LGl1encephalitis, emphasizing the differential diagnosis with prion disease (Creutzfeldt-Jakob disease).

**Teive & Arruda** wrote a historical note entitled "Cerebellum and cognition – Henrietta Leiner's contribution" in which they present the scientific contributions of Professor Henrietta Leiner, one of the pioneering scientists in the study of cognitive function of the cerebellum. **Sabatini et al.** reported the neuroimaging findings in a case of Wernicke's encephalopathy with chorea (Neuroimaging through clinical cases).

Finally, **Novaretti et al.** presented a case report of bipolar disorder as a harbinger of PD, and **Vasconcellos et al.** reported a case of Percheron thalamopeduncular syndrome with cervical dystonia.

**Egberto Reis Barbosa** Editor**Hélio A. G. Teive** Editor**Vitor Tumas** Editors

